# Understanding Eating Behavior during the Transition from Adolescence to Young Adulthood: A Literature Review and Perspective on Future Research Directions

**DOI:** 10.3390/nu10060667

**Published:** 2018-05-24

**Authors:** F. Marijn Stok, Britta Renner, Peter Clarys, Nanna Lien, Jeroen Lakerveld, Tom Deliens

**Affiliations:** 1 Department of Psychological Assessment and Health Psychology, University of Konstanz, P.O. Box 47, 78457 Konstanz, Germany; britta.renner@uni-konstanz.de; 2 Department of Movement and Sport Sciences, Faculty of Physical Education and Physiotherapy, Vrije Universiteit Brussel, 1050 Brussels, Belgium; pclarys@vub.ac.be (P.C.); tom.deliens@vub.be (T.D.); 3 Department of Nutrition, University of Oslo, 0315 Oslo, Norway; nanna.lien@medisin.uio.no; 4 Department of Epidemiology and Biostatistics & the EMGO Institute for Health and Care Research, VU University Medical Center, 1081 BT Amsterdam, The Netherlands; j.lakerveld@vumc.nl

**Keywords:** life transitions, eating behavior, behavior change, weight change, emerging adulthood, literature review

## Abstract

Introduction: Eating behavior often becomes unhealthier during the transition from adolescence to young adulthood, but not much is known about the factors that drive this change. We assess the available evidence on this topic through a literature review and pay special attention to the research designs employed in the studies available as well as the modifiability of the factors investigated in previous research. Method: We systematically conducted a scoping review by searching literature published in or after 2000 in three databases that described one or more factors associated with eating behavior or changes in eating behavior during the transition from adolescence to adulthood in the general population. Our search identified eighteen articles meeting these inclusion criteria. The socio-ecological DONE (Determinants of Nutrition and Eating) framework, a recently developed dynamic framework of factors shaping dietary behavior, was used to structure and categorize the factors identified. Results: Most factors identified in the literature were individual-level factors (67%) such as food beliefs, time constraints, and taste preferences; on the other hand, interpersonal-level factors (e.g., social support), environmental-level factors (e.g., product characteristics) and policy-level factors (e.g., market regulations) have been reported on less extensively. Furthermore, most factors discussed in the literature have been classified in the DONE framework as not easily modifiable. Moreover, previous studies largely used static research designs and focused primarily on one specific population (US freshmen). Discussion: This systematic scoping review identified several gaps in the available literature that hinder insight into the drivers of eating behavior (change) during the transition from adolescence to young adulthood. There is an urgent need for research on broader populations, employing dynamic repeated-measures designs, and taking modifiability of factors into account.

## 1. Introduction

The transition from adolescence to young adulthood, typically thought to occur between 18 and 25 years of age [1] is a time of transformation. Important and life-changing transitions typically occur during this period, such as leaving high school to start college or working life, and leaving the parental home to establish an independent living arrangement. As such, this life phase is marked by adaptation to changing physical and social contexts, substantial increases in independence and autonomy, and a development of one’s individuality and identity [1,2,3]. This transitional life stage was, until recently, generally considered to be a low-risk period in life, in which people generally enjoy good health. This point of view has recently become more nuanced, however, and emerging adulthood has gained recognition as a period marked by critical health risks. The main reason for this is that emerging adulthood seems to be associated with an increased risk for weight gain [4,5] and it has been argued that the increase in overweight and obesity prevalence is in fact larger among emerging adults than in any other age group (for an overview, see [5]). The heightened risk of weight gain in this stage of life seems to be due, at least in part, to the fact that weight-related behavioral patterns are subject to substantial change throughout this period [5,6,7,8].

Corroborating this assumption, it has been shown that changes in eating behavior (such as increasing intake of snacks, more frequent breakfast skipping, and decreasing intake of fruits and vegetables) are important contributors to weight gain during the transition from adolescence to young adulthood [4,5,6,7]. These findings have recently instigated the development and implementation of various interventions aimed at promoting healthy eating specifically during this life stage [9]. Crucially, however, relatively little attention has been paid until now to the factors driving eating behavior during this transitional life stage [5], whereas one might argue that the development of effective interventions in fact hinges on the identification of the mechanisms driving the shift toward unhealthier eating behavior in emerging adults. 

### A Focus on Shaping Factors

Whereas substantial evidence shows that emerging adulthood carries an increased risk for unhealthy eating [4,5,6,7,8], not all emerging adults show a shift towards unhealthier eating or show it to the same degree [6]. This individual differentiation indicates that unhealthier eating behavior during the transition from adolescence to young adulthood is not normative or unavoidable. We posit that, in order to better tailor interventions and to more effectively prevent unhealthy changes in eating behaviors during this critical transition period, it is of crucial importance to gain a better understanding of the factors driving eating behavior during this life stage (see also [5]). This requires increased focus on the precursors of eating in emerging adulthood, that is, on the factors that shape eating behavior during this transitional period. Through identifying the driving factors behind the eating behaviors of emerging adults, we can identify entry points for intervention and policy alike. To date, research examining the factors shaping eating behavior in the transition from adolescence to adulthood is limited [5]. Nevertheless, there have been several attempts to gain insight into these factors. 

In order to synthesize the available evidence about the factors shaping eating behavior during the transition from adolescence to young adulthood, we conducted a scoping review of currently available literature. This review accumulates evidence available about the factors driving eating behavior in emerging adulthood and organizes the factors that have been identified in the literature according to a socio-ecological framework of determinants of eating behavior, the multidisciplinary DONE (determinants of nutrition and eating) framework [10]. Furthermore, in order to inform future research and intervention planning, the type of research design employed in each study included in the review is recorded and the modifiability of the factors (that is, the extent to which the factors are theoretically amenable to change [10,11,12]) identified in the review is discussed. To our knowledge, this review constitutes the first systematic attempt at synthesizing the factors shaping eating behavior in emerging adulthood that have been uncovered in the literature thus far. Based on the findings from the scoping review, limitations of the research that has been conducted on this topic until now are identified, highlighting the main gaps in the current body of knowledge. 

## 2. Materials and Methods

A scoping literature search was systematically performed in three databases: Web of Science (WoS), PubMed (PM), and PsycInfo (PI). A scoping literature review [13,14] has an exploratory character aimed at getting a grasp of the body of evidence available on a broad topic, so as to provide an overview of the type, quantity and quality of research available on a given topic and to identify future research needs [13,15,16]. Another characteristic of scoping reviews is that although the process of literature gathering tends to be rapid (e.g., in terms of number of databases searched and extensiveness of search strings used), the search process is still fully systematic and transparent. Following The Cochrane Collaboration’s PICOS guidelines [17] for the conduction of a systematic literature search, a focused question of interest was first established. We decided to focus our search on four concepts and formed search strings for each concept (see Table 1 for exact search terms): Participants (concept 1), Outcomes (concept 2), Setting (concept 3) and Study design (concept 4). Because including the latter two concepts simultaneously into one four-layered search yielded very few hits, we chose to conduct two separate three-layered searches, including concepts 1 and 2 plus either concept 3 or concept 4. We restricted our search to articles published in English in or after the year 2000. The literature search was conducted in 2017. 

The search was conducted by the first (FMS) and last (TD) authors, with TD searching one database (WoS) and FMS searching two databases (PM and PI). FMS and TD also screened and selected the records that came out of these respective databases, discussing any record that raised questions until agreement was reached. Remaining doubts or disagreements were further discussed and resolved with the second author (BR). Both authors involved in the search and selection process (FMS and TD) have extensive background in performing systematic literature searches, having been previously involved in multiple successful systematic literature reviews [18,19,20,21].

We maintained the following inclusion criteria: (1) The article had to mention at least one factor that was associated with eating behavior. For the purposes of this article, we were interested in actual eating behaviors such as food intake (frequency, quantity), food choice, and portion size as outcome measures. We excluded articles focusing on outcomes such as attitudes or intentions regarding food or eating, articles focused on weight change rather than eating behavior, as well as articles focusing on problematic eating behaviors as disordered or pathological eating, emotional eating, and night-time eating; (2) Participants in the study had to come from the general population, so studies on specific subgroups (e.g., medical students, international students) were excluded; (3) In order to ensure that studies truly focused on determinants of eating behavior during the transition period from adolescence to young adulthood, we set the criterion that the time frame of prospective quantitative studies should include the transition period. Quantitative studies employing a sample of college or university students were therefore included only when the sample consisted of freshmen; those incorporating a larger age range (such as those including students from all undergraduate study years) were excluded. Qualitative studies were included if they were aimed specifically at the transitional period (retrospectively), regardless of when the study was conducted. 

**Figure 1:**
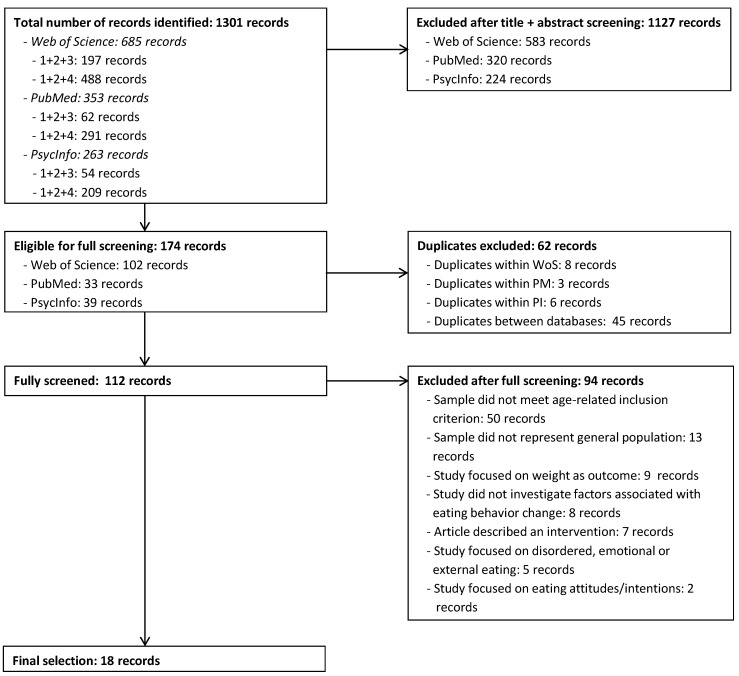
PRISMA flow diagram of the identification, screening and selection of articles for the review.

Figure 1 provides a PRISMA flow diagram [22] of the identification, screening and selection process. Combined across all three search engines and both combinations of search terms, the literature search yielded a total of 1301 hits that were screened based on title and abstract. This screening was done separately for each three-layered search and for each database (meaning that records that appeared in multiple databases and/or in both three-layered searchers were screened multiple times). A total of 1127 records were excluded in this phase, leaving 174 articles eligible for full screening. Duplicates were excluded at this stage. Duplicates occurred both within one database, if both three-layered searches yielded one and the same hit (*N* = 8 for WoS; *N* = 3 for PM; *N* = 6 for PI) as well as between the databases (*N* = 45). After removing these duplicates, articles were fully screened. A total of 94 articles were excluded after full screening. Exclusion of articles happened for seven reasons (in case multiple reasons for exclusion held true for one article, it was counted under the first reason mentioned here): (1) the sample did not meet the age-related inclusion criterion that was set (*N* = 50); (2) the sample represented a specific subgroup rather than the general population (*N* = 13); (3) the study outcomes were related to weight (change) rather than eating behavior (*N* = 9); (4) the article described changes in eating behavior but did not investigate any factors associated with such changes (*N* = 8); (5) the article described an intervention (*N* = 7); (6) the study outcomes were related to emotional, external, or disordered eating (*N* = 5); (7) the study outcome was attitudes or intentions about eating, rather than actual eating behavior (*N* = 2). After full screening of the remaining articles, a set of eighteen unique articles [23,24,25,26,27,28,29,30,31,32,33,34,35,36,37,38,39,40] remained (see Table 2). 

**Figure 2:**
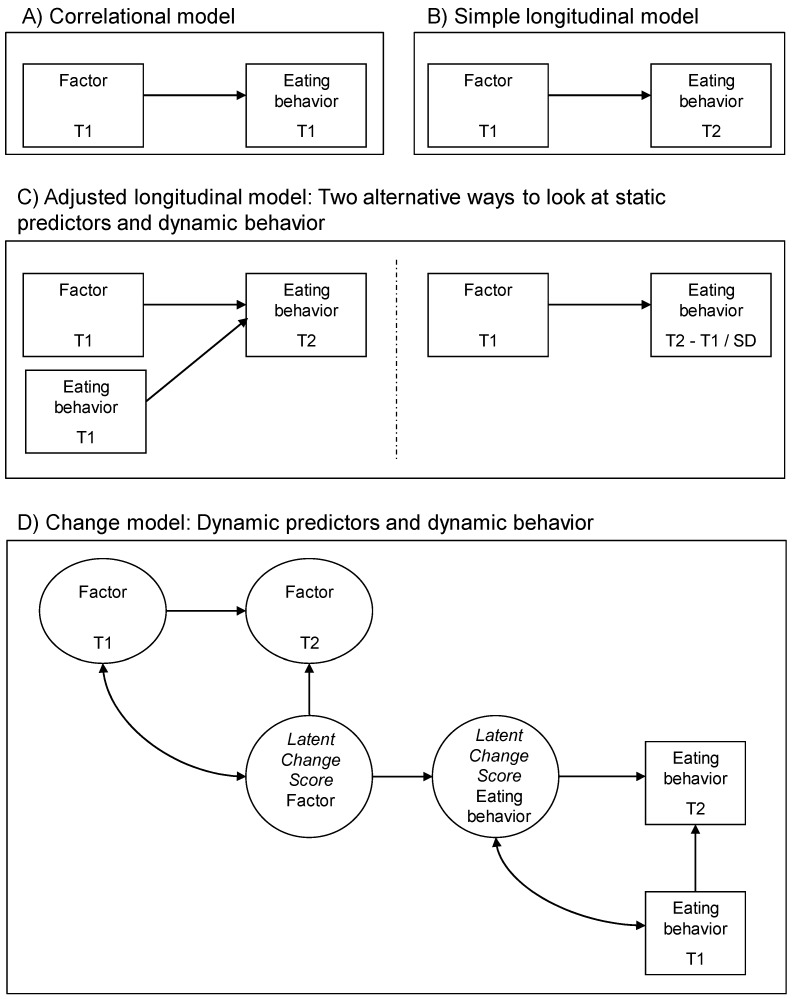
Example models of static and dynamic research designs. Note: T = time point; SD = standard deviation. This figure provides a visualization of static (panels **A**–**C**) and dynamic (panel **D**) research designs. Each design investigates a relation between a shaping factor and an outcome—for the current purposes, this would be an outcome related to eating behavior. The designs become progressively more complex: (Panel **A**) describes a cross-sectional design where a factor at time 1 is associated with an outcome at the same time point. (Panel **B**) describes a simple longitudinal design where a factor at time 1 predicts an outcome at a later time, time 2. (Panel **C**) describes an adjusted longitudinal design wherein a factor at time 1 still predicts an outcome at a later time, but now taking into account the baseline (time 1) level of that outcome. (Panel **D**) describes a dynamic research design including two measurement moments for both factor and outcome, such that it can be determined whether changes in the factor predict changes in the outcome. This figure is adapted from Renner et al., 2008 [41] and is reproduced with the first author’s permission.

In order to assess to which extent the studies included in the review are able to capture the dynamic process by which changes in shaping factors affect subsequent changes in eating behavior, the study design of each article included in the review was recorded. This was done using the classification proposed by Renner and colleagues [41] depicted in Figure 2, which distinguishes between four different kinds of research designs. Each design investigates a relation between a shaping factor and an outcome. The designs as described by Renner and colleagues become progressively more complex: in a correlational design, a factor at time 1 is associated with an outcome at the same time point. In a simple longitudinal design, a factor at time 1 predicts an outcome at a later time point. In an adjusted longitudinal design, a factor at time 1 still predicts an outcome at a later time point, but now the baseline (time 1) level of that outcome is taken into account. Crucially, each of these designs is static because they do not account for changes in the factor. In order to truly understand what drives change in the outcome, it is necessary to study changes in factors and see if such changes are followed by changes in outcomes, i.e., to determine whether changes in the factor predict changes in the outcome. The final research design described by Renner and colleagues, the dynamic research design, accomplishes this by including two measurement moments for both shaping factor and outcome. 

The multidisciplinary DONE (Determinants of Nutrition and Eating) framework [10] was employed to structure and categorize the factors identified in these articles, and to facilitate systematic interpretation and analysis. The DONE framework is a dynamic framework of the shaping factors of diet-related outcomes across the life course. It has been developed by a workgroup of almost 90 experts from a wide variety of disciplines in the context of the European research project and knowledge hub DEDIPAC (Determinants of Diet and Physical Activity, [42,43]). The factors in the framework were nominated both bottom-up (based on the workgroup members’ expertise and experience) as well as top-down (based on literature review), with the ultimate aim of collecting all potential shaping factors of diet-related outcomes. The framework currently comprises 449 determinants and has a four-level structure. Determinants are first sorted into four main socio-ecological levels of influence (individual, interpersonal, environment, and policy). Each socio-ecological level is further subdivided into two additional lower levels of sub-categories, called stem-categories and leaf-categories. For example, the shaping factor ‘having a food allergy’ is categorized into the individual socio-ecological level. Within that level, it is further categorized into the stem-category ‘biological’ and, within that stem-category, it is further categorized into the ‘food-related physiology’ leaf-category. The first and last authors (FMS and TD) performed the first categorization of factors according to the DONE framework. Unclarities were resolved through mutual discussion between FMS and TD with assistance from BR. All authors inspected and agreed on the final categorization.

An important advantage of the framework is that the factors’ modifiability, relationship strength and population-level effect have been rated by almost 200 experts (both workgroup members as well as external experts). Together, these ratings provide an indication of priority for research for each of the factors included in the framework. For purposes of the current perspective, we will focus on the ratings of modifiability only. Possible modifiability scores range from 1 (low modifiability) to 3 (high modifiability). Average modifiability per stem-category (i.e., the most specific level of categorization of the framework) as judged by the experts involved in the development of the DONE framework, is used as an indicator for the modifiability of the specific factors identified in the current literature search. This rating indicates to what extent experts in the field consider it theoretically possible to modify the average determinant in this stem-category.

## 3. Results

The eighteen articles identified described results of original research on factors associated with (changes in) eating behavior during the transitional period occurring between adolescence and young adulthood. The fact that only eighteen articles were found that met the inclusion criteria corroborates the idea that there is a paucity of studies on this topic. Of the eighteen studies, twelve (67%) of the studies used a sample of college or university students [24,25,26,27,28,29,35,36,37,38,39,40], while six (33%) of the studies used a more general sample of young people [23,30,31,32,33,34]. Six articles (33%) were qualitative in nature [25,26,28,29,36,38], four (22%) were cross-sectional [24,27,35,37], and eight (44%) were longitudinal [23,30,31,32,33,34,39,40] (though it should be noted that five of these stemmed from one project and were based on one and the same data set). Importantly, while qualitative and cross-sectional study designs can of course identify potentially relevant factors, by definition they do not allow the drawing of conclusions about whether any given factor truly shapes the *changes* in eating behavior that occur during the transition from adolescence to young adulthood. While longitudinal study designs are better equipped to do this, all but one of the longitudinal studies identified in the literature search (39% of the total number of articles) used a simple or adjusted longitudinal design [23,30,31,32,33,39,40] (investigating how a factor assessed at a first time point affected eating behavior assessed at a second time point). Most longitudinal studies thus did not include repeated measurements of both the shaping factor as well as the eating behavior, which, according to Renner and colleagues [41], would be the only way to plausibly establish that (changes in) the factor affected (changes in) the behavior. Only one of the longitudinal studies did employ such a design with repeated measurements of both factor and outcome [34]. 

One hundred and five distinct factors were described in the articles (see Supplemental File S1 available online for the complete list of factors). A large majority (*N* = 67; 64%) of the factors identified in the eighteen articles were individual-level factors. Within these individual-level factors, the majority of factors (*N* = 39) were psychological factors, which thus clearly and consistently emerged as drivers of eating behavior during emerging adulthood. The specific leaf-categories from which psychological factors were identified were ‘mood and emotions’ (e.g., boredom); ‘self-regulation’ (e.g., self-control); ‘health cognitions’ (e.g., healthy eating intentions); ‘food knowledge, skills and abilities’ (e.g., food preparation involvement); ‘food beliefs’ (e.g., perceived barriers to healthy eating); ‘food habits’ (e.g., past eating habits); and ‘weight control cognitions and behaviors’ (e.g., dietary restraint). Other individual-level factors were situational factors (*N* = 16; e.g., hunger, time constraints, television viewing, accessibility to a kitchen), biological factors (*N* = 8; e.g., taste preferences, health status) and demographic (*N* = 4, e.g., income) These factors generally had relatively low modifiability scores (average modifiability across the leaf-categories = 1.83 out of a maximum of 3.00), with modifiability judged slightly higher for factors from the ‘psychological’ and ‘situational’ stem-categories than for factors from the ‘biological’ and ‘demographic’ stem-categories. 

Fewer upstream factors were identified. At the interpersonal level, *N* = 18 factors were found (17%), which were sorted into the specific leaf-categories of ‘family food culture’ (e.g., family meal frequency); ‘household socio-economic status’ (e.g., family affluence); ‘social influence (e.g., peer pressure); ‘social support’ (e.g., parental support for healthy eating); ‘parental behaviors’ (e.g., parental control); and ‘cultural cognitions’ (e.g., socio-cultural norms and values). Modifiability of these factors was relatively low as well (average modifiability across the leaf-categories = 1.82 out of 3.00). At the environmental level, 18 factors were found (17%). Most of these environmental factors were related either to the product (*N* = 5, e.g., product taste, product convenience) or to the micro-environment (*N* = 10, e.g., availability of unhealthy food in the home, college campus food environment, lack of healthy food options on campus). At the meso- and macro-environmental level, on the other hand, only *N* = 3 factors were found (marketing strategies, media and advertising, and state of the local economy). Importantly, modifiability of factors at the environmental level was judged by the experts to be higher (average modifiability across the leaf-categories represented in the review = 2.15 out of 3.00). Finally, only *N* = 2 factors were identified at the policy level (2%), the most distant socio-ecological level in the DONE framework (food-related government policy, and market regulations). Modifiability of policy-level factors was judged to be moderate (modifiability of the leaf-category represented in the review = 2.11 out of 3.00). 

To summarize, our literature review identified eighteen articles describing factors shaping eating behavior change in emerging adulthood. Synthesis of these articles indicated that all but one of the studies described in these articles employed static research designs (i.e., qualitative, cross-sectional, simple longitudinal or adjusted longitudinal designs, see [41]). Only one study included in the review employed a dynamic research design. Also, most studies focused specifically on populations of college students. Moreover, taken together, the eighteen articles identified a total of 105 distinct factors shaping eating behavior changes in the transitioning period from adolescence to adulthood. Most of these factors were individual-level factors (mainly psychological factors); significantly fewer factors were identified at the interpersonal level and at the product-environmental and micro-environmental levels; and hardly any factors were identified at the meso-/macro-environmental and policy levels. Thus, with each further step ‘upstream’ on the socio-ecological ladder, fewer factors could be identified that have been studied in previous research. In addition, many of the factors that were frequently studied had relatively low modifiability scores. This was especially true for the individual-level and interpersonal-level factors that were identified in the articles. There was a tendency for factors at more distal socio-ecological levels (e.g., characteristics of the eating environment, exposure to food promotion, and governmental regulations) to score higher on modifiability. Yet, as described, these more upstream factors have been included in research less often.

## 4. Discussion

A scoping review of the available literature on the factors shaping eating behavior (change) during the transition from adolescence to young adulthood confirmed that there is a paucity of research on this topic; only eighteen articles were identified that reported on shaping factors of eating behavior in emerging adulthood. Synthesis of these articles indicated that most studies employed static research designs, mainly studied college students, and primarily investigated individual-level factors which had rather low modifiability—more upstream factors (which tend to have higher modifiability scores) were identified much less often. From this research synthesis, we thus identify several important limitations of the current body of research. In our perspective, three main issues are of relevance: firstly, the lack of studies using a design that can properly establish which factors truly affect eating behavior *change*; secondly, the lack of focus on the modifiability of the factors shaping eating behavior change during emerging adulthood; and thirdly, the strong focus on college freshmen as study populations.

### 4.1. Static Versus Dynamic Research Designs

Importantly, the nature and design of the studies that have been conducted to date are not ideally suited to determine which factors are systematically and strongly associated with changes in eating behavior throughout the transition. The scoping review revealed that previous quantitative studies have overwhelmingly employed static designs (see Figure 1 for an explanation of static and dynamic research designs). Such designs are not sensitive to the dynamic nature of eating behavior changes, which occur in different degrees from one individual to another. Some previous studies (e.g., [27]) have used cross-sectional designs, meaning that they can only provide an indication of a correlation between factor and outcome at one point in time; no conclusions can be drawn about the relevance of any given factor in shaping the changes in eating behavior that occur during the transition from adolescence to young adulthood. Other previous studies (e.g., [33]) included a longitudinal design. While this body of research certainly represents a step in the right direction, it still only provides ‘static’ observations, as it does not include a dynamic account of changes in both the factors which cause the behavior change and the outcome that is the actual behavior change, across multiple time points. In order to plausibly state that any given factor drives changes in eating behavior, it should be shown that variation in the factor is systematically followed by variation in eating behavior [41,44]. 

Thus, a study investigating the factors that drive eating behavior change in emerging adulthood should optimally include repeated measurements of both the hypothesized factors and the eating-related outcomes of interest, with measurements starting well before the transition, continue throughout, and end only after the transition is considered completed. Importantly, out of all the longitudinal studies identified in this scoping review, only one [34] employed a so-called dynamic approach as described above; the other seven longitudinal studies thus did not employ a research design that allows establishing which changes in shaping factors cause which changes in eating behavior during the transition period. Moreover, a considerable percentage of previous studies have been qualitative in nature (e.g., [29]). While qualitative studies are typically more sensitive to the dynamic nature of changes than static quantitative studies, they are of course limited by the lack of quantitative data to support the findings. Rather, these studies provide potential factors that should subsequently be studied in rigorous quantitative study designs. 

Interestingly, a review was recently conducted investigating the measurement of studies of dietary intake in the period from adolescence to adulthood [45]. That is, this review investigated the methods through which earlier studies have aimed to identify changes in eating behavior in emerging adults (thus not focusing on the factors shaping these changes). This study came to quite similar conclusions regarding future research directions, as the authors also indicate a lack of adequately assessed longitudinal data and posit that in order to further our understanding of dietary trajectories in this transitioning period, high-quality dietary assessment methods are required across longer time trajectories. Based on the findings from our current review, we would add to these conclusions that in order to also gain insight into the mechanisms behind the changes in eating behavior across emerging adulthood, dynamic repeated-measures designs (where both determining factors and outcomes are measured before, during and after the transition) are instrumental. To stimulate further progress in this vital area of research, longitudinal and dynamic designs are necessary, that are able to capture changes over time in factor and outcome alike, and that will thus allow us to shed light on the mechanisms driving the deteriorating eating behaviors of emerging adults. We propose that a complete research design investigating factors associated with changes in eating behavior during the transition from adolescence to young adulthood should comprise at least three measurement moments: before, during and after the transition. At each of these measurement moments, both factor and outcome should be assessed. 

### 4.2. Modifiability of Factors

One of the main reasons for attempting to identify shaping factors of eating behavior in emerging adults is to find leverage points for interventions. It has been argued that one of the crucial aspects to take into account when determining entry points for interventions is whether the factors identified are at all amenable to change or modifiable [10,11,12]. As has been previously advocated [10,11,12], when determining leverage points for interventions, health promotion experts should always take into account not only the strength of the relation between factor and outcome, but also the changeability of the factor, as this is a crucial aspect in determining their suitability as potential targets of interventions.

However, the current review indicates that previous research on eating behavior in emerging adulthood has not paid much attention to the question of modifiability. Moreover, many of the studies to date have focused on factors that, according to expert ratings available in the DONE framework [10], are not easily modifiable. This suggests that the factors that are most researched may not actually be the most strategic targets for interventions. It would be important to take such insights into account when planning future research on factors shaping eating behavior in emerging adults, so as to focus our research efforts in the most promising direction. The review also indicated that more upstream (e.g., environmental and policy) factors seem to be viable, changeable entry points for intervention, but that these types of factors have been largely overlooked in previous research efforts [23,24,25,26,27,28,29,30,31,32,33,34,35,36,37,38,39,40], which have focused predominantly on individual factors. The DONE framework [10] specifies a substantial number of modifiable factors on the interpersonal, environmental and policy levels of influence, but to date there is a lack of longitudinal research investigating the relationships between changes in these factors and changes in eating behavior in the transitional period from adolescence to young adulthood (although it should be acknowledged that, at the most upstream socio-ecological levels, in some cases it may become difficult to ensure sufficient variability in factors to employ such dynamic research designs). Including the core categories of the DONE framework in future research could facilitate employment of an integrated and dynamic approach, instead of focusing on individual cogs in the system. 

### 4.3. A Focus on US College Freshmen

Another issue is the fact that the majority of studies that have investigated eating behavior during the transition from adolescence to young adulthood were conducted in samples of US college freshmen. These studies can only provide a partial answer to the question of what factors drive eating behavior changes. A substantial number of adolescents do not transition from high school to college: about one-third of all students graduating from high-school in the US in 2017 is currently not enrolled in a college or university [46]; in the UK, more than 70% of 18-year-olds do not go directly into higher tertiary education after high-school [47]. These adolescents thus experience a very different transitioning context than those going to college or university; they may transition to e.g., working life, a different type of education, or a so-called ‘gap year’. Because of the differences in context, factors that shape eating behavior change in those emerging adults going into higher tertiary education may not necessarily be similar to factors shaping eating behavior in those who do not. Similarly, as there are large socio-cultural differences in the college freshmen experience between, for example, the US and Europe, results from the US cannot be automatically generalized to other countries. There is thus also a need for research on broader transitioning populations. Cohort studies may provide an excellent opportunity for such types of research. 

## 5. Strengths and Limitations

Strengths of the current review are that a systematic, transparent search strategy was employed to provide an overview of the current body of knowledge on the topic at hand. Using the DONE framework [10] allowed for a structured classification of the factors identified in the literature, and allowed us to investigate at which socio-ecological levels factors have been identified at which frequency. By recording the research designs employed in each study included in the review, and using an independently established model to classify these designs, we were able to judge the extent to which currently available studies are able to capture dynamic processes of change in eating behavior and its shaping factors. This has provided important information for future research directions. Using modifiability scores established by a large group of experts, we have been able to judge the extent to which the factors that have until now been identified as drivers of eating behavior change in emerging adulthood are theoretically amenable to change. This, too, has provided important insights for future research efforts, as well as for the identification of entry points for intervention and policies. Finally, the research team involved in this review is multidisciplinary, international, and has extensive experience in conducting systematic literature reviews. 

In terms of limitations, the review was set up as a scoping review. As such, we have to note the possibility that the search process may not have identified *all* articles available on this specific research topic, for example because the number of databases searched was limited and because we only searched for published, peer-reviewed articles in English published in or after the year 2000. The format of a scoping review matched our research aims to provide an overview of the quantity and quality of available studies and, especially, to give a perspective on future research directions [13,14,15,16]—yet, readers should take our chosen approach into account when interpreting the body of literature identified in the current review.

## 6. Conclusions

In order to learn which factors can be considered viable targets for policy and interventions, it is necessary to achieve a good understanding of (a) which factors are strongly and systematically associated with changes in eating behavior; and (b) which of these factors are modifiable (presuming that changing these factors will subsequently change the behavior). Therefore, two crucial aspects for new research in this area are the use of dynamic research designs capable of capturing changes in both shaping factor and behavioral outcome, and a focus on modifiability of the shaping factors under investigation. Scholars should focus not only on individual-level factors, but pay substantial attention as well to more upstream factors, as there are indications (e.g., [10]) that factors at more distal socio-ecological levels tend to be more modifiable according to experts. Lack of attention for these aspects may hamper our ability to reach a comprehensive understanding of eating behavior in emerging adulthood. This, in turn, may impede the development of optimally effective and targeted interventions, as a more complete and accurate understanding of the phenomenon of (changes in) eating behavior during the transition from adolescence to young adulthood is a necessary precondition for doing so. Furthermore, the field would benefit from the employment of more diverse research populations. Emerging adulthood is a crucial phase for health, as it is frequently paired with a shift towards unhealthy eating behaviors, which in turn may lead to negative health consequences. Achieving a better understanding of the modifiable factors that drive unhealthy eating behaviors in this transitional life stage is essential for the development of tailored interventions.

## Figures and Tables

**Table 1:** Overview of search terms employed.

Search Terms	
1.	(student* or freshman or freshmen or college* or universit* or “higher education” or “late adolesc*” or “young adult*” or “emerging adult*” or “18-2*” or “17-2*” or “16-2*” or “new adult*”).ti.
2.	(nutrit* or diet* or eat* or food* or fruit* or vegetable* or sugar* or fat* or soda* or “soft drink*” or “sugar sweetened beverage*” or intake or snack*).ti.
3.	(transition* or change or “school to work” or “school to college” or period* or critical or phase* or stage*).ti.
4.	(determinant* or correlat* or associat* or mediat* or moderat* or predict*).ti.
5.	1 and 2 and 3 (first search performed in each database)
6.	1 and 2 and 4 (second search performed in each database)

**Table 2:** List of articles included in the scoping review.

Ref #	Article	Country	Study Population	Description of Study	Study Design
[23]	Barr-Anderson et al., 2009	US	from middle school and high school to 17–20-year olds (mean age at follow-up = 17.2 ± 0.6 years and 20.5 ± 0.8 years for younger and older cohorts, respectively)	television viewing as a predictor of FV, whole grain, calcium, trans fat, fried food, fast food, snacks, and SSB intake	simple longitudinal
[24]	Brunstrom et al., 2008	UK	first-year undergraduate students (mean age = 18.7 ± 0.8 years)	determinants of portion size (of snacks, side dishes, and main meals)	cross-sectional
[25]	Cluskey & Grobe, 2009	US	college students (mean age = 19.0 years)	determinants of eating behavior	qualitative
[26]	Deliens et al., 2014	Belgium	university students (mean age = 20.6 ± 1.7 years)	determinants of eating behavior	qualitative
[27]	Guagliardo et al., 201	France	first-year students (mean age = 19.5 years; range = 18–24 years)	eating at university canteen as predictor of FV, meat, fish, salt, fat, and fiber intake	cross-sectional
[28]	Kwok et al., 2016	Hong Kong	first-year students (age range = 18–24 years)	determinants of food choice	qualitative
[29]	LaCaille et al., 2011	US	college students (mean age = 19.3 ± 1.2 years)	determinants of eating behavior	qualitative
[30]	Larson et al., 2007b	US	adolescence to young adulthood (mean age at follow-up = 20.4 years)	family meal frequency as a predictor of main meal frequency and FV, whole grain, calcium and SD intake	adjusted longitudinal
[31]	Larson et al., 2008a	US	adolescence to young adulthood (mean age at follow-up = 20.4 ± 0.8 years)	correlates of FV intake	adjusted longitudinal
[32]	Larson et al., 2008b	US	adolescence to young adulthood (mean age at follow-up = 20.5 ± 0.9 years)	correlates of fast food intake	adjusted longitudinal
[33]	Larson et al., 2009	US	adolescence to young adulthood (mean age at follow-up = 20.5 ± 0.8 years)	correlates of calcium and dairy intake	adjusted longitudinal
[34]	Lipsky et al., 2015	US	adolescence to young adulthood (mean age at baseline = 16.3 years)	determinants of whole grain, SSB, snacks, and FV intake	dynamic longitudinal
[35]	Lloyd-Richardson et al., 2008	US	college freshmen (mean age = 18.6 ± 0.04 years)	alcohol consumption as a predictor of overeating and unhealthy eating	cross-sectional
[36]	Nelson et al., 2009	US	freshmen and sophomore college students (mean age = 19.4 years; range = 18–21 years)	determinants of dietary intake	qualitative
[37]	Poulos & Pasch, 2015	US	college freshmen (mean age = 18.7 years)	energy drink consumption as a predictor of (diet) soda, milk, snacks, frozen food, FV, and fast food intake and breakfast and restaurant frequency	cross-sectional
[38]	Strong et al., 2008	US	first and second year college students (mean age = 18.3 ± 0.1 years)	determinants of eating behavior	qualitative
[39]	Tomasone et al., 2015	Canada	first-year undergraduate students (mean age =17.8 ± 0.5 years)	trait self-control, attitudes, subjective norms, perceived behavioral control and intentions as predictors of FV intake	simple longitudinal
[40]	Wengreen & Moncur, 2009	US	first-year college students (aged 18–19 years)	changes in weight, dietary intake, and other health-related behaviors, and correlations between these	adjusted longitudinal *

Note: FV = fruits and vegetables; SD = soft drinks; SSB = sugar-sweetened beverages. When ‘determinants’ or ‘correlates’ is not further specified, a broad spectrum of factors was assessed. When ‘eating behavior’, ‘food choice’ or ‘dietary intake’ is not further specified, no specific categories were assessed in the study (this is typical for qualitative designs). Several of the studies in this table assessed additional outcome variables that were not relevant to the current study purpose; these are not described in this table. Research designs are specified according to the different categories depicted in Figure 2. * This study is in fact set up as a dynamic longitudinal design, yet the analyses presented do not account for changes in eating behavior; the model presented therefore remains a static model.

## Supplementary Materials

The following are available online at http://www.mdpi.com/2072-6643/10/6/667/s1. File S1: Factors associated with (changes in) eating behavior during the transition from adolescence to young adulthood.

## References

[1] Arnett J.J. (2010). Adolescence and Emerging Adulthood: A Cultural Approach.

[2] Erikson E.H. (1968). Identity, Youth, and Crisis.

[3] Gall T.L., Evans D.R., Bellerose S. (2000). Transitions to first-year university: Patterns of change in adjustment across life domains and time. J. Soc. Clin. Psychol..

[4] Deforche B., Van Dyck D., Deliens T., De Bourdeaudhuij I. (2015). Changes in weight, physical activity, sedentary behaviour and dietary intake during the transition to higher education: A prospective study. Int. J. Behav. Nutr. Phys. Act..

[5] Nelson M.C., Story M., Larson N.I., Neumark-Sztainer D., Lytle L.A. (2008). Emerging adulthood and college-aged youth: An overlooked age for weight-related behavior change. Obesity.

[6] Finlayson G., Cecil J., Higgs S., Hill A., Hetherington M. (2012). Susceptibility to weight gain. Eating behaviour traits and physical activity as predictors of weight gain during the first year of university. Appetite.

[7] Niemeier H.M., Raynor H.A., Lloyd-Richardson E.E., Rogers M.L., Wing R.R. (2006). Fast food consumption and breakfast skipping: Predictors of weight gain from adolescence to adulthood in a nationally representative sample. J. Adolesc. Health.

[8] Larson N.I., Neumark-Sztainer D., Hannan P.J., Story M. (2007). Trends in adolescent fruit and vegetable consumption, 1999–2004: Project EAT. Am. J. Prev. Med..

[9] Laska M.N., Pelletier J.E., Larson N.I., Story M. (2012). Interventions for weight gain prevention during the transition to young adulthood: A review of the literature. J. Adolesc. Health.

[10] Stok F.M., Hoffmann S., Volkert D., Boeing H., Ensenauer R., Stelmach-Mardas M., Kiesswetter E., Weber A., Rohm H., Lien N. (2017). The DONE framework: The DONE framework: Creation, evaluation, and updating of an interdisciplinary, dynamic framework 2.0 of determinants of nutrition and eating. PLoS ONE.

[11] Booth S.L., Sallis J.F., Ritenbaugh C., Hill J.O., Frank L.D. (2001). Environmental and societal factors affect food choice and physical activity: Rationale, influences, and leverage points. Nutr. Rev..

[12] Green L.W., Kreuter M.W. (1991). Health Promotion Planning: An Educational and Environmental Approach.

[13] Anderson S., Allen P., Peckham S. (2008). Asking the right questions: Scoping studies in the commissioning of research on the organization and delivery of health services. Health Res. Policy Syst..

[14] Davis K., Drey N., Gould D. (2009). What are scoping studies? A review of the nursing literature. Int. J. Nurs. Stud..

[15] Armstrong R., Hall B.J., Doyle J., Waters E. (2011). Cochrane Update: ‘Scoping the scope’ of a cochrane review. J. Public Health.

[16] Grant M.J., Booth A. (2009). A typology of reviews: An analysis of 14 review types and associated methodologies. Health Inf. Libr. J..

[17] Higgins J.P.T., Green S. (2011). Cochrane Handbook for Systematic Reviews of Interventions.

[18] Stok F.M., De Vet E., De Ridder D.T.D., John B.F. (2016). The potential of peer social norms to shape food intake in adolescents and young adults: A systematic review of effects and moderators. Health Psychol. Rev..

[19] Deliens T., Van Crombruggen R., Verbruggen S., De Bourdeaudhuij I., Deforche B., Clarys P. (2016). Dietary interventions among university students: A systematic review. Appetite.

[20] De Ridder D.T.D., Lensvelt-Mulders G., Finkenauer C., Stok F.M., Baumeister R.F. (2012). Taking stock of self-control: A meta-analysis of how trait self-control relates to a wide range of behaviors. Pers. Soc. Psychol. Rev..

[21] Hohenauer E., Stoop R., Clarys P., Clijsen R., Deliens T., Taeymans J. (2018). The effect of pre-exercise cooling on performance characteristics: A systematic review and meta-analysis. Int. J. Clin. Med..

[22] Moher D., Liberati A., Tetzlaff J., Altman D.G., The PRISMA Group (2009). Preferred Reporting Items for Systematic Reviews and Meta-Analyses: The PRISMA statement. BMJ.

[23] Barr-Anderson D.J., Larson N.I., Nelson M.C., Neumark-Sztainer D., Story M. (2009). Does television viewing predict dietary intake five years later in high school students and young adults?. Int. J. Behav. Nutr. Phys. Act..

[24] Brunstrom J.M., Rogers P.J., Pothos E.M., Calitri R., Tapper K. (2008). Estimating everyday portion size using a ‘method of constant stimuli’: In a student sample, portion size is predicted by gender, dietary behaviour, and hunger, but not BMI. Appetite.

[25] Cluskey M., Grobe D. (2009). College weight gain and behavior transitions: Male and female differences. J. Am. Diet. Assoc..

[26] Deliens T., Clarys P., De Bourdeaudhuij I., Deforche B. (2014). Determinants of eating behaviour in university students: A qualitative study using focus group discussions. BMC Public Health.

[27] Guagliardo V., Lions C., Darmon N., Verger P. (2011). Eating at the university canteen. Associations with socioeconomic status and healthier self-reported eating habits in France. Appetite.

[28] Kwok S.T., Capra S., Leveritt M. (2016). Factors influencing changes in eating patterns among Hong Kong young adults transitioning to tertiary education. Asia Pac. Public Health.

[29] LaCaille L.J., Dauner K.N., Krambeer R.J., Pedersen J. (2011). Psychosocial and environmental determinants of eating behaviors, physical activity, and weight change among college students: A qualitative analysis. J. Am. Coll. Health.

[30] Larson N.I., Neumark-Sztainer D., Hannan P.J., Story M. (2007). Family meals during adolescence are associated with higher diet quality and healthful meal patterns during young adulthood. J. Am. Diet. Assoc..

[31] Larson N.I., Neumark-Sztainer D.R., Harnack L.J., Wall M.M., Story M.T., Eisenberg M.E. (2008). Fruit and vegetable intake correlates during the transition to young adulthood. Am. J. Prev. Med..

[32] Larson N.I., Neumark-Sztainer D., Harnack L., Wall M., Story M., Eisenberg M.E. (2009). Calcium and dairy intake: Longitudinal trends during the transition to young adulthood and correlates of calcium intake. J. Nutr. Educ. Behav..

[33] Larson N.I., Neumark-Sztainer D.R., Story M.T., Wall M.M., Harnack L.J., Eisenberg M.E. (2008). Fast food intake: Longitudinal trends during the transition to young adulthood and correlates of intake. J. Adolesc. Health.

[34] Lipsky L.M., Haynie D.L., Liu D., Chaurasia A., Gee B., Li K., Iannotti R.J., Simons-Morton B. (2015). Trajectories of eating behaviors in a nationally representative cohort of US adolescents during the transition to young adulthood. Int. J. Behav. Nutr. Phys. Act..

[35] Lloyd-Richardson E.E., Lucero M.L., DiBello J.R., Jacobson A.E., Wing R.R. (2008). The relationship between alcohol use, eating habits and weight change in college freshmen. Eat Behav..

[36] Nelson M.C., Kocos R., Lytle L.A., Perry C.L. (2009). Understanding the perceived determinants of weight-related behaviors in late adolescence: A qualitative analysis among college youth. J. Nutr. Educ. Behav..

[37] Poulos N.S., Pasch K.E. (2009). Energy drink consumption is associated with unhealthy dietary. behaviours among college youth. Perspect. Publ. Health.

[38] Strong K.A., Parks S.L., Anderson E., Winett R., Davy B.M. (2008). Weight gain prevention: Identifying theory-based targets for health behavior change in young adults. J. Am. Diet. Assoc..

[39] Tomasone J.R., Meikle N., Bray S.R. (2015). Intentions and trait self-control predict fruit and. vegetable consumption during the transition to first-year university. J. Am. Coll. Health.

[40] Wengreen H.J., Moncur C. (2009). Change in diet, physical activity, and body weight among young-adults during the transition from high school to college. Nutr. J..

[41] Renner B., Hankonen N., Ghisletta P., Absetz P. (2012). Dynamic psychological and behavioral changes in the adoption and maintenance of exercise. Health Psychol..

[42] Lakerveld J., van der Ploeg H.P., Kroeze W., Ahrens W., Allais O., Andersen L.F., Cardon G., Capranica L., Chastin S., Donnelly A. (2014). Towards the integration and development of a cross-European research network and infrastructure: The DEterminants of DIet and Physical ACtivity (DEDIPAC) Knowledge Hub. Int. J. Behav. Nutr. Phys. Act..

[43] Brug J., van der Ploeg H.P., Loyen A., Ahrens W., Allais O., Andersen L.F., Cardon G., Capranica L., Chastin S., De Bourdeaudhuij I. (2017). Determinants of diet and physical activity (DEDIPAC): A summary of findings. Int. J. Behav. Nutr. Phys. Act..

[44] Bauman A.E., Sallis J.F., Dzewaltowski D.A., Owen N. (2002). Toward a better understanding of the influences on physical activity: The role of determinants, correlates, causal variables, mediators, moderators, and confounders. Am. J. Prev. Med..

[45] Winpenny E.M., Penney T.L., Corder K., White M., van Sluijs E.M.F. (2017). Change in diet in the period from adolescence to early adulthood: A systematic scoping review of longitudinal studies. Int. J. Behav. Nutr. Phys. Act..

[46] United States Bureau of Labor Statistics (2017). Economic News Release: College Enrollment and Work Activity of Recent High School and College Graduates Summary.

[47] United Kingdom Department for Education (2017). National Statistics: Participation Rates in Higher Education: 2006 to 2016.

