# Supplementary material for: Understanding Eating Behavior during the Transition from Adolescence to Young Adulthood: A Literature Review and Perspective on Future Research Directions
Source: Nutrients. 2018 May 24;10(6):667. doi: 10.3390/nu10060667 (PMC6024552; doi:10.3390/nu10060667)
Supplement: Supplementary file 1 [file nutrients-10-00667-s001.pdf]

Supplemental file 1: Factors associated with (changes in) eating behavior during the transition from adolescence to young adulthood

Note: This appendix lists all the factors associated with (changes in) eating behavior as identified in our literature search. The factors are sorted using the DEDIPAC DONE framework (9) structure. In the DONE framework, four main socio-ecological levels of influence are distinguished (individual, interpersonal, environment, policy), each further subdivided into stem-categories. Within each stem-category, several more specific leaf-categories of determinants are distinguished. The DONE framework provides an indication of the modifiability of each of these categories (based on expert ratings, see (9) for more information), which is also provided here. This rating indicates to what extent experts in the field consider it theoretically possible to modify the average determinant in this stem-category (so note that the ratings are not based on the exact same factors identified in this scoping review, but on similar determinants belonging to the same specific leaf-category within the framework). Possible modifiability scores range from 1.00 (lowest modifiability) to 3.00 (highest modifiability).

#	Name of factor	Source article	DONE framework: Socio-ecological level of influence and stem-category	DONE framework: Specific leaf-category	DONE framework: Modifiability of specific category
1	lactose intolerance	Larson, 2009	**Individual**; Biological	Food-Related Physiology	1.53
2	metabolism	Deliens, 2014			
3	BMI	Lipsky, 2015		Anthropometrics	1.80
4	weight gain	Wengreen, 2009			
5	taste preferences	Deliens, 2014; Larson, 2008a; Larson, 2009		Sensory Perception	1.74
6	physical and mental health status	Kwok, 2016		Physical Health	1.60
7	vitality	Deliens, 2014			
8	ethnicity	Lipsky, 2015	**Individual**; Demographic	Cultural Characteristics	1.46
9	gender	Brunstrom, 2008; Lipsky, 2015			
10	living off campus; cooking and shopping for self	LaCaille, 2011		Situational Demographics	1.67
11	residency (living arrangement)	Deliens, 2014			
12	income	Kwok, 2016		Personal Socio-Economic Status	1.73
13	boredom	Nelson, 2009	**Individual**; Psychological	Mood And Emotions	1.70
14	mood	Deliens, 2014			
15	self-control	LaCaille, 2011; Tomasone, 2015		Self-Regulation	1.80
16	self-discipline	Deliens, 2014			
17	time management skills	Cluskey, 2009; Nelson, 2009			
18	healthy eating inentions	Tomasone, 2015		Health Cognitions	2.09
19	intrinsic motivation	Cluskey, 2009			
20	motivation	LaCaille, 2011			
21	autonomy in food choices	Kwok, 2016		Food Knowledge, Skills And Abilities	2.27
22	dietary knowledge	Deliens, 2014			
23	food preparation involvement	Cluskey, 2009; Larson, 2008a			
24	self-efficacy for healthy eating	Larson, 2008a; Larson, 2008b; Larson, 2009; Strong, 2008			
25	beliefs, norms and values regarding food	Deliens, 2014		Food Beliefs	2.16
26	perceived barriers to healthy eating	Larson, 2008a; Larson, 2009			
27	perceived benefits of healthy eating	Larson, 2008a; Strong, 2008			
28	perceived financial barriers to healthy eating	Strong, 2008			
29	perceived taste barriers to healthy eating	Larson, 2008b			
30	perceived time barriers to healthy eating	Larson, 2008b; Strong, 2008			
31	breakfast frequency	Larson, 2008a; Larson, 2009; Lipsky, 2015		Food Habits	1.91
32	dinner frequency	Larson, 2008a			
33	eating in front of the television	Lipsky, 2015			
34	eating out for variety	Nelson, 2009			
35	eating routines	Cluskey, 2009			
36	energy drink frequency	Poulos, 2015			
37	fast food frequency	Larson, 2008a; Larson, 2009			
38	frequency of eating at university canteens	Guagliardo, 2011			
39	lunch frequency	Larson, 2008b			
40	past eating habits	Deliens, 2014			
41	snack frequency	Larson, 2008a; Larson, 2008b; Larson, 2009			
42	soft-drink frequency	Larson, 2009			
43	willingness-to-pay	Nelson, 2009			
44	body image	Deliens, 2014		Weight-Control Cognitions And Behaviors	1.94
45	desire to gain weight	LaCaille, 2011			
46	dietary restraint	Brunstrom, 2008			
47	dieting	Brunstrom, 2008			
48	health concerns	Kwok, 2016; Larson, 2008a; Larson, 2008b			
49	healthy weight control behaviors	Larson, 2008a; Larson, 2009; Strong, 2008			

50	self-concept	Deliens, 2014			
51	weight concerns	Larson, 2008a; Larson, 2009			
52	hunger	Brunstrom, 2008; Nelson, 2009	**Individual**; Situational	Hunger	1.83
53	alcohol consumption	LaCaille, 2011; Lloyd-Richardson, 2008; Nelson, 2009		Related Health Behaviors	2.18
54	anticipating alcohol consumption	Nelson, 2009			
55	physical activity	Deliens, 2014; Lipsky, 2015			
56	television viewing	Barr-Anderson, 2009; Larson, 2008a; Larson, 2008b; Larson, 2009			
57	accessibility to a full kitchen	Nelson, 2009		Situational And Time Constraints	2.02
58	availability and accessibility of cooking supplies	Deliens, 2014; LaCaille, 2011			
59	convenience of meal preparation	Deliens, 2014			
60	daily rhythm/structure	Deliens, 2014			
61	distance between home and school	Kwok, 2016			
62	exam time	Deliens, 2014			
63	relative priority of meal preparation	Deliens, 2014			
64	stress	Deliens, 2014; Kwok, 2016; Nelson, 2009			
65	studying	Nelson, 2009			
66	time constraints	Deliens, 2014; LaCaille, 2011; Kwok, 2016; Nelson, 2009			
67	university lifestyle	Deliens, 2014			
68	food habits of the family	Kwok, 2016	**Interpersonal**; Social	Family Food Culture	1.86
69	family food education	Kwok, 2016			
70	family meal frequency	Larson, 2007; Larson, 2008a; Larson, 2009; Lipsky, 2015			
71	family affluence	Lipsky, 2015; Kwok, 2016		Household Socio-Economic Status	1.42
72	food insecurity	Larson, 2009			
73	being member of a student society	Deliens, 2014		Social Influence	2.00
74	influence of friends	Kwok, 2016; Strong, 2008			
75	peer pressure	Cluskey, 2009; Deliens, 2014			
76	socializing	Nelson, 2009			
77	social norms	Kwok, 2016			
78	parental support for healthy eating	Larson, 2008a		Social Support	2.07
79	peer support for healthy eating	Larson, 2008a; Larson, 2008b; Larson, 2009			
80	receiving food from family	Nelson, 2009			
81	social support	Cluskey, 2009; Deliens, 2014; LaCaille, 2011; Strong, 2008			
82	parental control	Deliens, 2014		Parental Behaviors	1.79
83	parenting style	Deliens, 2014			
84	culture	Kwok, 2016	**Interpersonal**; Cultural	Cultural Cognitions	1.80
85	socio-cultural norms and values	Deliens, 2014			
86	product taste	Kwok, 2016	**Environment**; Product	Intrinsic Product Attributes	2.12
87	appeal of food products	Deliens, 2014		Extrinsic Product Attributes	2.31
88	price of food	Cluskey, 2009; Deliens, 2014; Kwok, 2016			
89	price of healthy foods	LaCaille, 2011			
90	product convenience	Kwok, 2016			
91	home unhealthy food availability	Larson, 2008a; Larson, 2008b	**Environment**; Micro	Home Food Availability And Accessibility	2.20
92	campus food environment	Cluskey, 2009; LaCaille, 2011		Eating Environment	2.41
93	characteristics of the meal plans	LaCaille, 2011			
94	eating in all-you-can-eat cafeteria	LaCaille, 2011			
95	accessibility of food	Strong, 2008		Environment Food Availability And Accessibility	2.04
96	availability and accessibility of foods	Deliens, 2014			
97	availability of food on campus	Nelson, 2009			
98	lack of food outlet variety	Nelson, 2009			
99	lack of healthy options on campus	LaCaille, 2011			
100	mealtime milk availability	Larson, 2009			
101	marketing strategies (cheap deals)	Nelson, 2009	**Environment**; Meso & Macro	Exposure To Food Promotion	2.21
102	media and advertising	Deliens, 2014; Kwok, 2016			
103	state of the local economy	Kwok, 2016		Market prices	1.89
104	food-related government policy	Kwok, 2016	**Policy**; Government	Governmental Regulations	2.11
105	market regulations	Deliens, 2014			
